# Comparison of Cox Model Methods in A Low-dimensional Setting with Few Events

**DOI:** 10.1016/j.gpb.2016.03.006

**Published:** 2016-05-17

**Authors:** Francisco M. Ojeda, Christian Müller, Daniela Börnigen, David-Alexandre Trégouët, Arne Schillert, Matthias Heinig, Tanja Zeller, Renate B. Schnabel

**Affiliations:** 1 Department of General and Interventional Cardiology, University Heart Center Hamburg-Eppendorf, 20246 Hamburg, Germany; 2 German Center for Cardiovascular Research (DZHK), Hamburg/Kiel/Luebeck, Germany; 3 Sorbonne Universités, Université Pierre et Marie Curie Paris 06, Institut National pour la Santé et la Recherche Médicale (INSERM), Unité Mixte de Recherche en Santé (UMR_S) 1166, F-75013 Paris, France; 4 Institute for Cardiometabolism and Nutrition (ICAN), F-75013 Paris, France; 5 Institut für Medizinische Biometrie und Statistik, Universität zu Lübeck, Universitätsklinikum Schleswig-Holstein, Campus Lübeck, 23562 Lübeck, Germany; 6 Institute of Computational Biology, German Research Center for Environmental Health, Helmholtz Zentrum München, 85764 Neuherberg, Germany

**Keywords:** Proportional hazards regression, Penalized regression, Events per variable, Coronary artery disease

## Abstract

Prognostic models based on survival data frequently make use of the Cox proportional hazards model. Developing reliable Cox models with few events relative to the number of predictors can be challenging, even in low-dimensional datasets, with a much larger number of observations than variables. In such a setting we examined the performance of methods used to estimate a Cox model, including (i) full model using all available predictors and estimated by standard techniques, (ii) backward elimination (BE), (iii) ridge regression, (iv) least absolute shrinkage and selection operator (lasso), and (v) elastic net. Based on a prospective cohort of patients with manifest **coronary artery disease** (CAD), we performed a simulation study to compare the predictive accuracy, calibration, and discrimination of these approaches. Candidate predictors for incident cardiovascular events we used included clinical variables, biomarkers, and a selection of genetic variants associated with CAD. The penalized methods, *i.e.*, ridge, lasso, and elastic net, showed a comparable performance, in terms of predictive accuracy, calibration, and discrimination, and outperformed BE and the full model. Excessive shrinkage was observed in some cases for the penalized methods, mostly on the simulation scenarios having the lowest ratio of a number of events to the number of variables. We conclude that in similar settings, these three penalized methods can be used interchangeably. The full model and backward elimination are not recommended in rare event scenarios.

## Introduction

The applications of prognostic models, that is, models that predict the risk of a future event, include among others [1]: (i) informing individuals about a disease course or the risk of developing a disease, (ii) guiding further treatment decisions, and (iii) selecting patients for therapeutic research. Prognostic models derived using time-to-event (or survival) data often make use of the Cox proportional hazards model. Thernau and Grambsch [2] describe this model as the “workhorse of regression analysis for censored data”. When the number of events is small relative to the number of variables, the development of a reliable Cox model can be difficult. This can be challenging even in a low-dimensional setting where the number of predictors is much smaller than the number of observations. Existing rules of thumb are based on the number of events per variable (EPV), which is recommended to be between 10 and 20 [3], [4]. When performing variable selection, these EPV rules are applied to the number of candidate variables considered, not just those in the final model [3], [4]. Penalized regression methods that shrink the regression coefficients towards 0 are an option in a rare event setting, which may effectively increase the EPV [5], thus producing better results. Examples of these methods include ridge regression [6], the least absolute shrinkage and selection operator (lasso) [7], and the elastic net [8], which is a combination of the former two. Backward elimination (BE) is another widely used method [9] that seemingly reduces the number of predictors by applying *P* values and a significance level α to discard predictors (*α* = 0.05 is often used).

Our aim in this work was to compare, in a low EPV and low-dimensional setting, the performance of different approaches to computing the Cox proportional hazards model. We consider the following methods: (i) full model, computed using all predictors considered via maximization of the partial log-likelihood (termed “full” model), (ii) BE with significance levels *α* = 0.05 and *α* = 0.5 (BE 0.05 and BE 0.5), (iii) ridge, (iv) lasso, and (v) elastic net (for simplicity termed “elastic” thereafter).

## Results

### Simulation results

Simulations were used to compare different methods based on a prospective cohort study of patients with manifest coronary artery disease (CAD) [10]. Two main scenarios were considered: (1) clinical variables relevant to CAD such as age, gender, body mass index (BMI), high density lipoprotein (HDL) over low density lipoprotein (LDL) cholesterol ratio, current smoking, diabetes, and hypertension, as well as blood-based biomarkers such as C-reactive protein (CRP) and creatinine as predictors; and (2) information on 55 genetic variants in addition to the variables used in scenario 1. These variants represented either loci that have been previously shown to be associated, at the genome-wide significance level, with CAD, or recently-identified CAD loci [11]. Baseline characteristics are shown in Table S1. There are 1731 participants involved, with a median age of 63 years and 77.6% male. Table S2 provides information of the genetic variants used. The median follow-up was 5.7 years. In each scenario, a Weibull ridge model was fitted in the cohort. Each fitted model was considered the true model and was used to simulate the survival time. Censored Weibull quantile–quantile (Q–Q) plots of the models’ exponentiated residuals are shown in Figure S1. Deviations from the Weibull distribution are observed in both scenarios.

Cox proportional hazards models were calculated on the simulated datasets using the different methods considered (full model, BE, ridge, lasso, and elastic net) for EPV equal to 2.5, 5, and 10, respectively. BE 0.05 selected no variable in 64% (scenario 1) and 62% (scenario 2) of the simulations performed with EPV = 2.5. For the same EPV, BE 0.5 selected no variable in 18% and 10% of the simulations for scenarios 1 and 2, respectively. This resulted in a model that predicted the same survival probability for all individuals in the dataset (this model is basically a Kaplan–Meier estimator). The same occurred for BE with other EPV values and also for the lasso (32% and 25%) and the elastic net (8% and 2%) with EPV = 2.5. The ridge method also produced constant predictions (10% and 4% of the simulations, EPV = 2.5) as a consequence of shrinking the coefficients too strongly (in all cases where the elastic net gave constant predicted survival probabilities it was equal to or very close to the ridge model in the sense that elastic net mixing parameter was zero or almost zero). Consequently, the computation of the calibration slope and the concordance becomes impossible.

The calibration slope could not be calculated either, when a model assigned a predicted survival probability of 1 to at least one individual. This occurred for the full model in 72 (EPV = 2.5) and 3 (EPV = 5) simulations in scenario 1, and in 12 simulations in scenario 2 (EPV = 2.5). BE and the penalized models (ridge, lasso, and elastic net) had 62 and 8 simulations, respectively, that predicted a survival probability of 1 (all of them in scenario 1). The root mean square error (RMSE) could be computed in all these cases. However for consistency, the results shown below only reported the RMSE for the simulations where the concordance and calibration slope could be computed. Table 1 gives the number of simulations used to compute RMSE, calibration slope, and concordance on each scenario.

**Figure 1:**
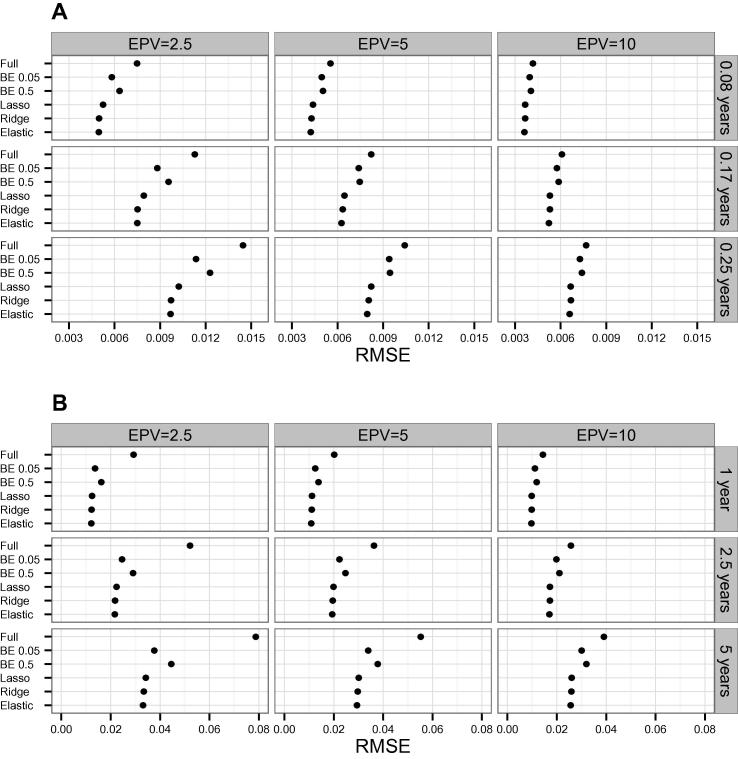
Average RMSEs across simulations for both scenarios using different models Average RMSEs of simulated datasets were calculated using different models in scenario 1 (**A**) and scenario 2 (**B**), respectively, with different EPV. The models examined include full model, BE with significance levels *α* = 0.05 and *α* = 0.5 (BE 0.05 and BE 0.5), ridge, lasso, and elastic net. Scenario 1 considers patients’ clinical variables relevant to CAD and blood-based biomarkers as predictors. Predicted event probabilities were computed at time points 0.08, 0.17, and 0.25 years, respectively. In scenario 2, information on 55 genetic variants is also considered besides the predictors used in scenario 1, while predicted event probabilities were computed at time points 1, 2.5, and 5 years, respectively. BE, backward elimination; RMSE, root mean square error; EPV, events per variable.

**Figure 2:**
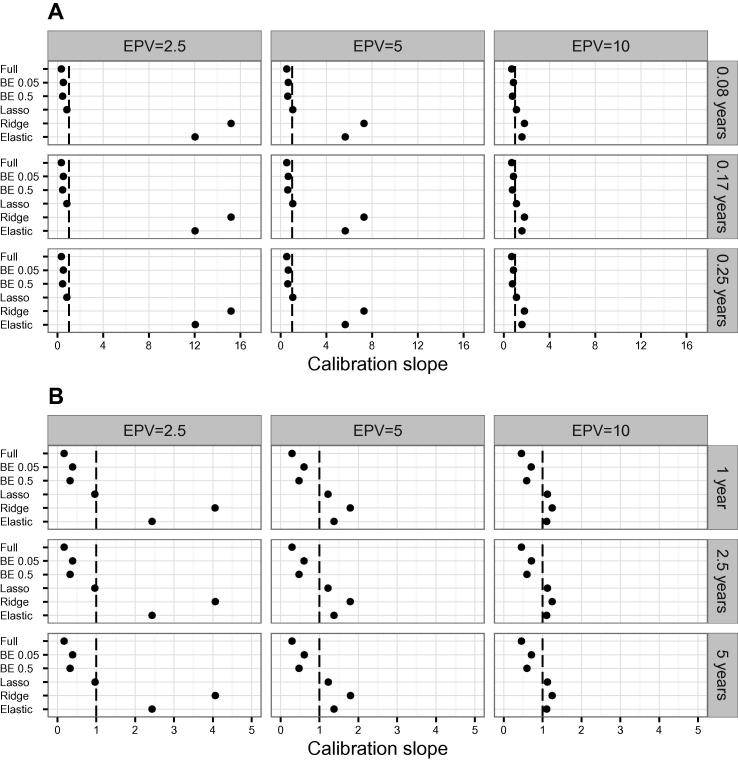
Average calibration slopes across simulations using different models Average calibration slopes of simulated datasets were calculated using different models in scenario 1 (**A**) and scenario 2 (**B**), respectively. Dashed line depicts ideal calibration slope of 1. See legend of Figure 1 for more details of the models used and the scenarios examined.

For both scenarios we found a decrease of the RMSE as the EPV increases (Figure 1). The penalized methods (ridge, lasso, and elastic net) have lower RMSE than the full model and the two BE variants considered. BE with a lower significance level (BE 0.05) showed a better RMSE than a higher significance level (BE 0.5) in our simulations. In both scenarios 1 (Figure 1A) and 2 (Figure 1B), the elastic net had the best RMSE, that is, the RMSE that was closer to zero.

Looking at the average of the calibration slope across the simulations (Figure 2), the lasso method showed the best performance, being of all the methods the one with an average calibration slope closest to the ideal value of 1. Here, we observed that the average calibration slope for the ridge and the elastic net for scenario 1 and EPV = 2.5 was above 10 (above 5 for EPV = 5, Figure 2A). A similar but less extreme average calibration slope was observed in scenario 2 (Figure 2B). These extreme average calibration slopes for the ridge and elastic net were caused by excessive shrinkage of the regression coefficients. The extreme calibration slopes corresponded almost exclusively to models where the elastic net equalled or was comparable to the ridge model.

Using a trimmed mean, 5% on each tail of the distribution, as a robust estimator of the mean, reduced the extreme calibration slopes in scenario 1 and EPV = 2.5 from approximately 15 to 9 for the ridge and from 12 to 6 for the elastic net. In scenario 2, the trimmed mean reduced the average calibration slope from approximately 4 to 2.26 for the ridge and from 2.4 to 1.12 for the elastic net (data not shown). Examining the median calibration slope (Figure S2), we observed that the ridge has the best calibration slope in both scenarios with EPV = 2.5 and the elastic net with EPV = 5. The distribution of the calibration slope across simulations is shown as boxplots in Figures S3 (scenario 1) and S4 (scenario 2). On the boxplots we see how the interquartile range (IQR) of the calibration slopes becomes narrower with increasing EPV, and that in both scenarios the ridge has the greatest calibration slope IQR for EPV = 2.5. For both the ridge and the elastic net, the increase in IQR with the decreasing EPV is proportionally larger on the 75th percentile-median difference, than in the median-25th percentile difference. A particular simulation in scenario 2 with EPV = 2.5 that produced extreme calibration slopes was examined. The calibration slopes for this simulation were 22 for the elastic net and 52.5 for the ridge. A scatterplot of the points (log odds) used to compute the calibration slope is shown in Figure S5. Here we observed that the range of the estimated log odds of event is much shorter than that of the true log odds, indicating that too much shrinkage was applied.

**Figure 3:**
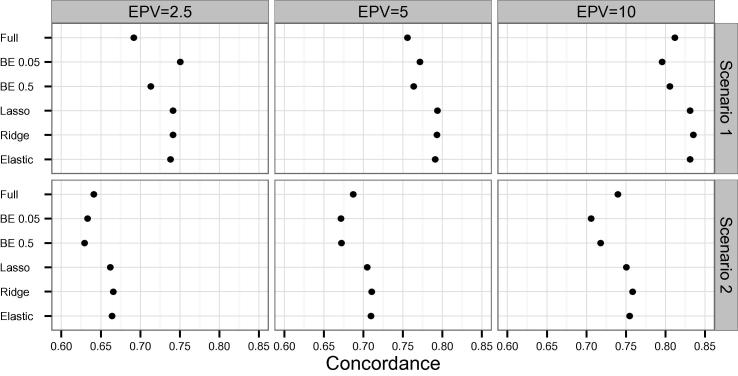
Average concordance across simulations using different models Average concordance of simulated datasets was calculated using different models in scenario 1 (**A**) and scenario 2 (**B**), respectively. See legend of Figure 1 for more details of the models used and the scenarios examined.

In both scenarios and all EPV values tested, the concordance was higher for the 3 penalized methods considered, except scenario 1 with EPV = 2.5, for which BE 0.05 had the highest concordance (Figure 3). In those cases for which the penalized methods showed better discrimination, either lasso or ridge had the highest concordance.

### BE and ridge

To further explore the methods considered, a hybrid method was considered, where BE was followed by an application of ridge regression, that is, the coefficients of the variables selected by BE were shrunk using ridge. Both BE 0.05 and BE 0.5 were examined. The results showed that RMSE of both BE 0.05 and BE 0.5 was improved by the application of ridge (Figure S6), but it was still higher than that when using ridge, lasso, or elastic net alone. With the application of ridge, both the average and the median calibration slope of BE came closer to the ideal value of 1 (Figures S7 and S8), whereas the concordance of BE (Figure S9) improved only slightly.

### Additional simulations

The three penalized methods considered have a tuning parameter, which gives the amount of shrinkage that is applied to the regression coefficients. The elastic net has an additional tuning parameter which determines how close the elastic net fit is to the lasso or ridge fit. These tuning parameters were selected in our simulations by 10-fold cross-validation. We next explored the sensitivity of the simulation results (RMSE, calibration slope, and concordance) for the penalized methods to the number of folds used in the cross-validation during the selection of tuning parameters. In particular, we wanted to examine whether the extreme calibration slopes observed in some of the simulations were attributed to the method used to select the tuning parameters. To do this, the simulations were repeated using 5-fold cross-validation (instead of 10-fold cross-validation as was done in the analyses shown above). RMSE, calibration slope, and concordance were overall similar to the previous results using 10-fold cross-validation (data not shown), including the distribution of the calibration slopes, in particular, the extreme values observed in some simulations.

Further additional simulations were run for the penalized methods using the predictor variables to balance the 10-folds used in the cross-validation. The observations were clustered in 10 groups via K-means and then each of the 10-folds used was chosen randomly so that it would contain approximately one tenth of the individuals on each cluster. Here again, the results for the RMSE, calibration slope, and concordance were similar to those for the initial simulations using 10-fold cross-validation, including the extreme values for the calibration slopes observed in some simulations (results not shown).

### Application to clinical data

The different methods considered, to compute a Cox model, were applied to the clinical data that were used as the basis of our simulations. We used the same scenarios as in the simulations (which are defined in terms of the candidate predictors used). The regression coefficients for both scenarios considered are shown in Tables S3 and S4. In scenario 1 (EPV = 23.2), creatinine was selected by all models performing selection (BE 0.05, BE 0.5, lasso, and elastic net), representing the only predictor selected by BE 0.05. BE 0.5 additionally selected age and C-reactive protein. The lasso and elastic net selected, on top of these two, LDL/HDL ratio, hypertension, and gender. In scenario 2 (EPV = 3.3), creatinine was the only predictor selected by BE 0.05, while BE 0.5 selected age additionally. None of the 55 variants considered was selected by these two methods. Lasso and the elastic net selected the same number of variables (24), of which 23 variables were selected by both methods. To quantify the discrimination of the different models we used the C-index [12], which estimates the probability that for a pair of individuals the one with the longest survival has also the longest predicted survival probability. The C-index is an extension of the area under the Receiver Operating Characteristics (ROC) curve (AUC) and has a similar interpretation [13]. In scenario 1, the full model had a C-index of 0.599 (Table 2). The highest C-index (0.601) was attained using ridge, followed by the elastic net and lasso (0.600). For scenario 2, the highest C-index was attained by the ridge (0.607), followed by the lasso (0.603) and the full model (0.601), while the elastic net had a C-index of 0.600. Both BE regressions considered had C-indices ⩽ 0.577. The BE C-indices improved slightly after applying ridge regression.

The full model had the calibration slope further away from the ideal value of 1 in both scenarios considered (0.868 and 0.5, respectively). The best calibration slope was achieved in scenario 1 by the lasso (1.012), followed by the combinations of BE 0.05 and BE 0.5 with the ridge (0.974 and 0.960, respectively), the elastic net (1.05), and the ridge method (1.065). The fact that these calibration slopes for the penalized methods were higher than 1 indicates that slightly too much shrinkage was applied by these three methods. In scenario 2, the best calibration slope was produced by the elastic net, followed by the lasso and ridge. Both BE methods had a calibration slope less than 0.65, indicating overfitting. The BE calibration slope was improved after applying ridge regression.

## Discussion

In this work we aimed to compare methods to compute a proportional hazards model in a rare event low-dimensional setting. Applying simulations based on a dataset of patients with manifest CAD, we compared the full model that used all predictors, BE with *α* = 0.05 or *α* = 0.5, ridge regression, lasso, and elastic net. The penalized methods, *i.e.*, ridge, lasso, and elastic net, outperformed the full model and BE, Nonetheless, there is no single penalized method that performs best for all metrics and both scenarios considered. BE performance was improved by shrinking the selected variable coefficients with ridge regression; however, this hybrid method was not better than ridge regression, lasso, or elastic net alone.

Ambler et al. [14] observed that the lasso and the ridge for Cox proportional hazards models have not been compared often in a low-dimensional setting. Porzelius et al. [15] investigated several methods that are usually applied in high-dimensional settings and produced sparse model fits, including the lasso and elastic net, in a low-dimensional setting, via simulations. They found the overall performance was similar in terms of sparseness, bias, and prediction performance, and no method outperforms the others in all scenarios considered. Benner et al. [16] found on their simulations that the lasso, ridge, and elastic net had an overall similar performance in low-dimensional settings. Ambler et al. [14], whose approach we follow in this paper, compared the models considered here on two datasets. They also studied the non-negative garrotte and shrank the coefficients of the full model by a single factor (estimated by bootstrap [17]), but they did not examine the elastic net. In their simulations, the ridge method performed better, except that lasso outperformed ridge for the calibration slope. The full model and BE performed the worst on low EPV settings. They recommend the ridge method, except when one is interested in variable selection where lasso would be better. They also observed that in some cases the ridge shrunk the coefficients slightly too much. Lin et al. [18] compared Cox models estimated by maximization of the partial likelihood, Firth’s penalized likelihood [19] and using the Bayesian approaches. They focused on the estimation of the regression coefficients and the coverage of their confidence intervals. They recommend using Firth’s penalized likelihood method when the predictor of interest is categorical and EPV < 6. Firth’s method was originally proposed as a solution to the problem of ‘monotone likelihood’ that may occur in datasets with low EPVs and that causes the standard partial likelihood estimates of the Cox model to break down.

In our simulations, there was no clear-cut winner, but certainly the penalized methods (ridge, lasso, and elastic net) performed better than the full model and BE. The elastic net showed the best predictive accuracy and all three penalized methods considered had comparable discrimination. In some of our simulations, the penalized methods shrunk the coefficients too much (in some cases extremely setting them to zero, including the ridge), even though the “true” model was being fitted. This behavior was observed both when using 10-fold and 5-fold cross-validation to select the tuning parameters of the penalized approaches and even after attempting to balance the folds based on the predictors. This suggests, as it was also pointed out previously [14], that more work should be done in developing methods to select the tuning parameters of the penalized approaches. Van Houwelingen et al. [20] describe a strategy involving penalized Cox regression, via the ridge, that can be used to obtain survival prognostic models for microarray data. In the first step of this approach, the global test of association [21] is applied and ridge regression is used only if the test is significant. Even though this approach is suggested in a high-dimensional setting, applying this global test on a low-dimensional setting before applying a penalized approach may help identify situations, where a penalized method may apply excessive shrinkage.

In our clinical dataset application on the scenario that included clinical variables, biomarkers, and genetic variants, the three penalized methods also had a comparable performance in terms of calibration and discrimination and showed better calibration than the full model and BE, in line with our simulation results.

Some limitations apply to our study. First, the Cox models received as input all variables used in the true underlying models to simulate the data, that is, there were no noise predictors. This may have given an unfair advantage to ridge regression which does penalization but not variable selection like the lasso or elastic net. Second, all simulations are based on a single clinical cohort, which may be representative of other cohorts, but we cannot compare, the similarity or dissimilarity of the observed simulation results in other datasets. Third, we examined only on the Cox proportional hazards model and did not consider alternative approaches to prognostic models for survival data like full parametric approaches or non-parametric ones (*e.g.*, survival random forest [22]). Future work will address some of these limitations on other datasets and using non-parametric models.

## Conclusion

All three methods using penalization, *i.e.*, ridge, lasso, and elastic net, provided comparable results in the setting considered and may be used interchangeably in a low EPV low-dimensional scenario if the goal is to obtain a reliable prognostic model and variable reduction is not required. If variable selection is desired, then the lasso or the elastic net can be used. Since too much shrinkage may be applied by a penalized method, it is important to inspect the fitted model to look for signs of excessive shrinkage. In a low EPV setting, the use of the full model and BE is discouraged, even when the coefficients of variables selected by BE are shrunk with ridge regression. This study adds new information to the few existing comparisons of penalized methods for Cox proportional hazards regression in low-dimensional datasets with a low EPV.

## Materials and methods

### Data

Athero*Gene* [10] is a prospective cohort study of consecutive patients with manifest CAD and at least one stenosis of 30% or more present in a major coronary artery. For the present study we focus on the combined outcome of non-fatal myocardial infarction and cardiovascular mortality. Time to event information was obtained by regular follow-up questionnaires and telephone interviews, and verified by death certificates and hospital or general practitioner charts.

Genotyping was performed in individuals of European descent only using the Genome-Wide Human SNP 6.0 Array (Affymetrix, Santa Clara, USA). The Markov chain haplotyping algorithm (MaCH v1.0.18.c) [23] was used to impute untyped markers. The 1000 Genomes Phase I Integrated Release Version 2 served as reference panel for the genotype imputation. For the present study we use 55 genetic variants (51 SNPs and 4 indels). These variants are taken from the CAD genome-wide association meta-analysis performed by the CARDIoGRAMplusC4D Consortium [11]. Using an additive genetic model, these variants represent the lead CARDIoGRAMplusC4D variants on 47 (out of 48) loci previously identified at genome-wide significance and 8 novel CAD loci found by this consortium. Out of the 48 loci examined [11], rs6903956 was not nominally significant and is not used in our analyses. All SNPs and indels are used as allele dosages, that is, the expected number of copies of specified allele is used in the analyses.

After exclusion of missing values, the dataset consists of 1731 individuals, 209 incident events and a median follow-up time of 5.7 years (with a maximum of 7.6 years).

### Design of simulations

We adopted the simulation design used by Ambler and colleagues [14] by considering two main scenarios. For scenario 1, we consider clinical variables (age, gender, BMI, HDL over LDL cholesterol ratio, current smoking, diabetes, and hypertension) and blood-based biomarkers (C-reactive protein and creatinine) as predictors. For scenario 2, we added information on 55 genetic variants to these variables. On each scenario, we fit a Weibull ridge model from which we simulate the survival time using the methods of Bender and colleagues [24]. Since the fitted Weibull model is used to simulate the survival time, this model provides the data generating mechanism, and as such it plays the role of the true underlying model. The resulting values of the survival time are then right-censored with help of a uniform random variable *U* on the interval (0, *δ*), that is, if the simulated time exceeds *U*, the (censored) time is set to *U*. The *δ*s are chosen to achieve an EPV of 2.5, 5, or 10 (lower *δ* values produce a higher percentage of censored time and therefore fewer observed events). We generate 1000 simulated datasets. For each scenario and EPV, and on each one of them we fit a standard Cox model via partial likelihood, two BE models, with *α* = 0.05 and *α* = 0.5, a lasso model, a ridge model and elastic net model.

### Penalized models

The Cox proportional hazards model assumes the hazard as follows,

(1) $$h(t)=h_{0}(t)\text{exp}\left(\overset{p}{\underset{j=1}{\sum }}\beta_{j}x_{j}\right)$$

where (*x*_1_, *x*_2_, …, *x_p_*) is a vector of *p* predictor variables (*e.g.*, age, gender, and BMI) and *β*_1_, *β*_2_, …, *β_p_* are the corresponding regression coefficients, which are the weights given to each variable by the model. These coefficients are obtained by maximizing the partial log-likelihood function *l*(*β*), where *β* = *(β*_1_, *β*_2_, …, *β_p_*).

For fixed non-negative *λ*, maximization of the penalized partial log-likelihood function,

(2) $$\frac{2}{n}l(\beta )-\lambda \left(\alpha \overset{p}{\underset{j=1}{\sum }}|\beta_{j}|+\frac{1}{2}(1-\alpha )\overset{p}{\underset{j=1}{\sum }}\beta_{j}^{2}\right)$$

produces the regression coefficients of the elastic net. The parameter *λ* controls the amount of shrinkage applied to the coefficients, higher values of lambda corresponding to lower coefficients. The parameter *α* is the elastic net mixing parameter and changes between 0 and 1 [25], [26]. The lasso and ridge regression coefficients are obtained by setting *α* to 1 and 0 in Eq. (2), respectively, and maximizing the resulting expression.

### Selection of tuning parameters for penalized models

For the lasso and the ridge, 10-fold cross validation is used and the parameter that maximizes the cross-validated partial log-likelihood [27] is used as the corresponding penalization parameter. For the elastic net, we consider a course grid from 0 to 1 in steps of length 0.05 for the mixing parameter *α*. As for the lasso and ridge, the cross-validated partial likelihood is maximized.

Additional analyses were performed selecting the tuning parameters using (1) 5-fold cross validation and (2) 10-fold cross-validation. The folds for the latter were obtained as follows. The observations were clustered in 10 groups using the predictors and K-means [28]. Then each fold was chosen randomly so that it would contain approximately one tenth of the individuals on each cluster.

### Comparison of methods

The use of a Weibull model to generate the data allows us to compare the “true” survival probabilities $S_{i}(t)$ of the *i*th individual at time *t*, to the survival probabilities S^i(t) estimated by the different models we considered. To compare survival probabilities, we used the same metrics as described previously [14]. RMSE for predictive accuracy is calculated as follows.

(3) RMSE(t)=1n∑i=1nSi(t)-S^i(t)2,

For calibration, the calibration slope $\alpha_{1}$ is used, which is the slope of the model obtained by fitting a simple linear regression to $y=\log\left(\frac{1-S_{i}(t)}{S_{i}(t)}\right)$ and x=log1-S^i(t)S^i(t). Ideally $\alpha_{1}$ should be 1 (overfitting occurs if $\alpha_{1}<1$ and underfitting occurs if $\alpha_{1}>1$). For discrimination the concordance, the proportion of pairs of patients where individuals with the higher predicted event probability also have the higher “true” event probabilities is used. It has a similar interpretation as the C-index and is related to Kendall’s rank correlation *τ* [29] according to the formula τ=2(concordance-0.5). For the RMSE and calibration slope the predicted survival probabilities are computed at time points 0.08, 0.17, and 0.25 years, respectively, for scenario 1 and of 1, 2.5, and 5 years, respectively, for scenario 2. The concordance is computed for only one time point, since its value does not depend on the particular time point used to compute the predicted survival probabilities.

### Analysis of the clinical dataset

The methods considered were applied to the Athero*Gene* dataset [10]. As measures of performance, we computed the C-index C_τ_ [12] and the calibration slope. For the computation of the C-index, the first five years of the follow-up were used. Since estimating the performance of a model on the same dataset the model was developed may produce over-optimistic performance estimates, both the C-index and calibration slope were corrected for over-optimism with help of the 0.632 bootstrap estimator [30]. 1000 bootstrap replications were used in the correction.

### Software

All analyses were performed with R Version 3.2.1. The *glmnet* package [25], [26] was used to fit the penalized Cox regressions (lasso, ridge, and elastic net). BE was performed with the package *rms* [4]. The *survival* package [2] was used to fit the standard Cox model. The *survC1* package was used to compute C_τ_.

## Authors’ contributions

FMO performed the simulations and data analyses. RBS provided the clinical perspective and information on the Athero*Gene* study. TZ performed genotyping and provided genetic information. CM and DB provided code and support for the data analyses. AS performed genotype calling and provided statistical advice. DAT performed genotype imputation. MH provided statistical advice. FMO drafted the manuscript. All authors critically revised and approved the final manuscript.

## Competing interests

The authors have declared no competing interests.

## Figures and Tables

**Table 1:** Number of simulations used when presenting results for different models out of a maximum of 1000 simulations

**Scenario**	**EPV**	**Full**	**BE 0.05**	**BE 0.5**	**Lasso**	**Ridge**	**Elastic**
1	2.5	928	345	785	681	903	913
1	5	997	649	945	871	976	983
1	10	1000	938	997	979	1000	1000
2	2.5	988	383	897	747	957	977
2	5	1000	784	992	938	994	997
2	10	1000	991	1000	998	1000	1000

*Note:* Presented in the table are the numbers of simulations where the model computed did not produce constant predictions nor predicted survival probabilities equal to 1. Scenario 1 candidate predictors include clinical variables and biomarkers. Scenario 2 candidate predictors include clinical variables, biomarkers, and genetic variants. BE, backward elimination; EPV, events per variable.

**Table 2:** C-indices and calibration slopes for clinical data example in both scenarios considered using different models

**Scenario**	**Measure**	**Full**	**BE 0.05**	**BE 0.5**	**BE 0.05 + Ridge**	**BE 0.5 + Ridge**	**Lasso**	**Ridge**	**Elastic**
1	C-index	0.599	0.586	0.596	0.586	0.596	0.600	0.601	0.600
2	C-index	0.601	0.574	0.577	0.574	0.578	0.603	0.607	0.600
1	Calibration slope	0.868	0.927	0.884	0.974	0.960	1.012	1.065	1.050
2	Calibration slope	0.500	0.649	0.583	0.708	0.645	0.861	1.162	0.885

*Note:* The C-indices and calibration slopes presented are corrected for over-optimism via the 0.632 bootstrap. BE 0.05 + ridge and BE 0.5 + ridge refer to ridge regression applied to the variables selected by BE 0.05 and BE 0.5, respectively. BE, backward elimination.

## Supplementary material

Supplementary Figure S1 **Censored Weibull Q**−**Q plot for residuals of Weibull ridge regression models used as the base of the simulations** Scenario 1 considers patients’ clinical variables relevant to CAD and blood-based biomarkers as predictors. In scenario 2, information on 55 genetic variants is also considered besides the predictors used in scenario 1. A Weibull regression model was fitted to the clinical data used as the basis of the simulations for each scenario. The residuals of this fit are then used to produce a Q–Q plot. Due to the censoring of the data, the points at which the quantiles of the theoretical distribution are computed are estimated using the Kaplan–Meier estimator. The residuals are exponentiated to be able to compare them against the Weibull distribution. Q–Q plot, quantile–quantile plot.

Supplementary Table S1 Baseline characteristics of patients in the Athero*Gene* study used in our simulations.

Supplementary Table S2 Information on genetic variants considered.

Supplementary Table S3 Regression coefficients for scenario 1 (EPV = 23.22) using different models.

Supplementary Table S4 Regression coefficients for scenario 2 (EPV = 3.27) using different models.

Supplementary Figure S2 **Median calibration slope across simulations using different models** Median calibration slopes of simulated datasets were calculated using different models in scenario 1 (A) and scenario 2 (B), respectively. Dashed line depicts ideal calibration slope of 1. The models examined include full model, BE with significance levels *α* = 0.05 and *α* = 0.5 (BE 0.05 and BE 0.5), ridge, lasso, and elastic net. BE, backward elimination; EPV, events per variable.

Supplementary Figure S3 **Calibration slope distribution across simulations for scenario 1 using different models** (A) Boxplots of calibration slopes. (B) Boxplots of calibration slopes with outliers removed to help visualization of quartiles. Predicted event probabilities were computed at time point 0.08 year. Gray dashed line depicts ideal calibration slope of 1.

Supplementary Figure S4 **Calibration slope distribution across simulations for scenario 2 using different models (**A) Boxplots of calibration slopes. (B) Boxplots of calibration slopes with outliers removed to help visualization of quartiles. Predicted event probabilities were computed at time point 1 year. Gray dashed line depicts ideal calibration slope of 1.

Supplementary Figure S5 **Scatter plot of log odds of event for exemplary simulation with extreme calibration slope** The true log odds of event was plotted against the estimated log odds of event. Results shown here are for exemplary simulation in scenario 2 with elastic net (left panel) and ridge regression (right panel). One year predicted event probabilities were computed at time point 1 year. Blue line depicts least squares regression line (*y* = 72.2 + 22 *x* and *y* = 176.7 + 52.5 *x* for elastic net and ridge, respectively). Dashed line depicts the *y* = *x* line, which corresponds to the ideal case in which the estimated log odds equals to the true log odds.

Supplementary Figure S6 **Average RMSEs across simulations including application of ridge regression to results of BE** Average RMSEs of simulated datasets were calculated using different models in scenario 1 (A) and scenario 2 (B), respectively. Other than full model, BE with significance levels *α* = 0.05 and *α* = 0.5 (BE 0.05 and BE 0.5), ridge, lasso, and elastic net, models examined also include application of ridge regression for BE 0.5 (BE 0.5 + Ridge) and BE 0.05 (BE 0.05 + Ridge). Predicted event probabilities were computed at time points 1, 2.5, and 5 years. BE, backward elimination; RMSE, root mean square error; EPV, events per variable.

Supplementary Figure S7 **Average calibration slopes across simulations including application of ridge regression to results of BE** Average calibration slopes of simulated datasets were calculated using different models in scenario 1 (A) and scenario 2 (B), respectively. Dashed line depicts ideal calibration slope of 1. See legend of Figure S6 for more details of the models used and the scenarios examined.

Supplementary Figure S8 **Median calibration slopes across simulations including application of ridge regression to results of BE** Median calibration slopes of simulated datasets were calculated using different models in scenario 1 (A) and scenario 2 (B), respectively. Dashed line depicts ideal calibration slope of 1. See legend of Figure S6 for more details of the models used and the scenarios examined.

Supplementary Figure S9 **Average concordance across simulations including application of ridge regression to results of BE** Average concordance of simulated datasets was calculated using different models in scenario 1 (A) and scenario 2 (B), respectively. See legend of Figure S6 for more details of the models used and the scenarios examined.
