# Supplementary figures and images for: Comparison of Cox Model Methods in A Low-dimensional Setting with Few Events
Source: Genomics Proteomics Bioinformatics. 2016 May 17;14(4):235–43. doi: 10.1016/j.gpb.2016.03.006 (PMC4996851; doi:10.1016/j.gpb.2016.03.006)

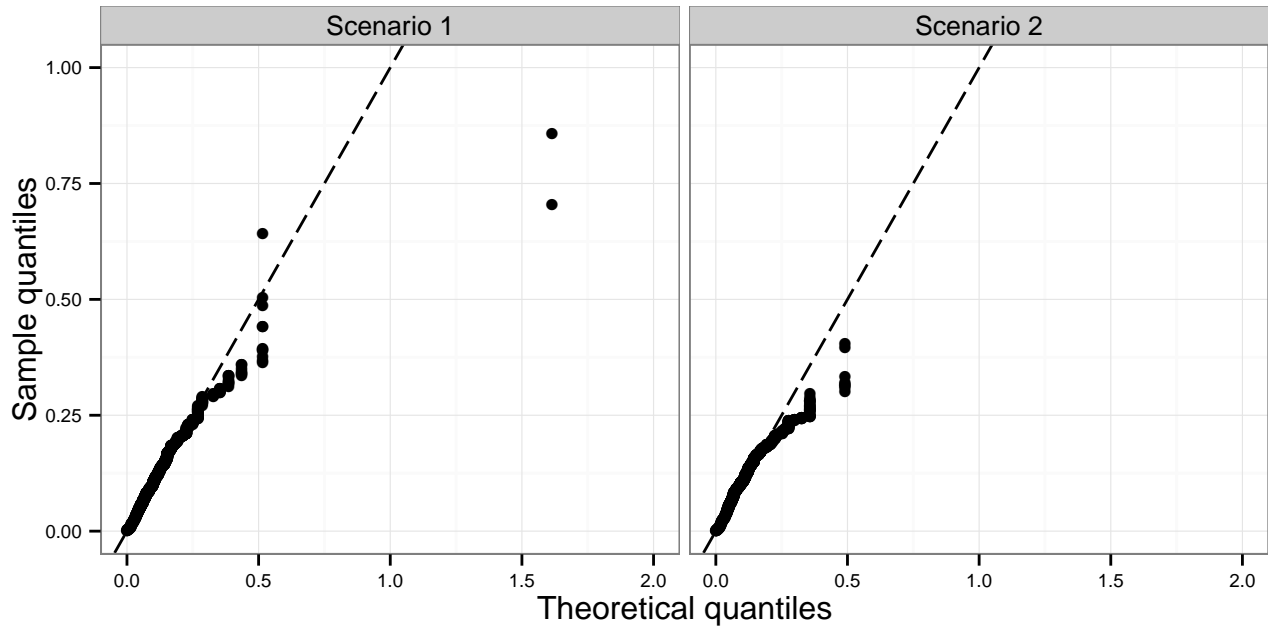

Supplement: Supplementary Figure S1 — Censored Weibull Q−Q plot for residuals of Weibull ridge regression models used as the base of the simulations Scenario 1 considers patients’ clinical variables relevant to CAD and blood-based biomarkers as predictors. In scenario 2, information on 55 genetic variants is also considered besides the predictors used in scenario 1. A Weibull regression model was fitted to the clinical data used as the basis of the simulations for each scenario. The residuals of this fit are then used to produce a Q–Q plot. Due to the censoring of the data, the points at which the quantiles of the theoretical distribution are computed are estimated using the Kaplan–Meier estimator. The residuals are exponentiated to be able to compare them against the Weibull distribution. Q–Q plot, quantile–quantile plot. [file mmc1.pdf]

**A**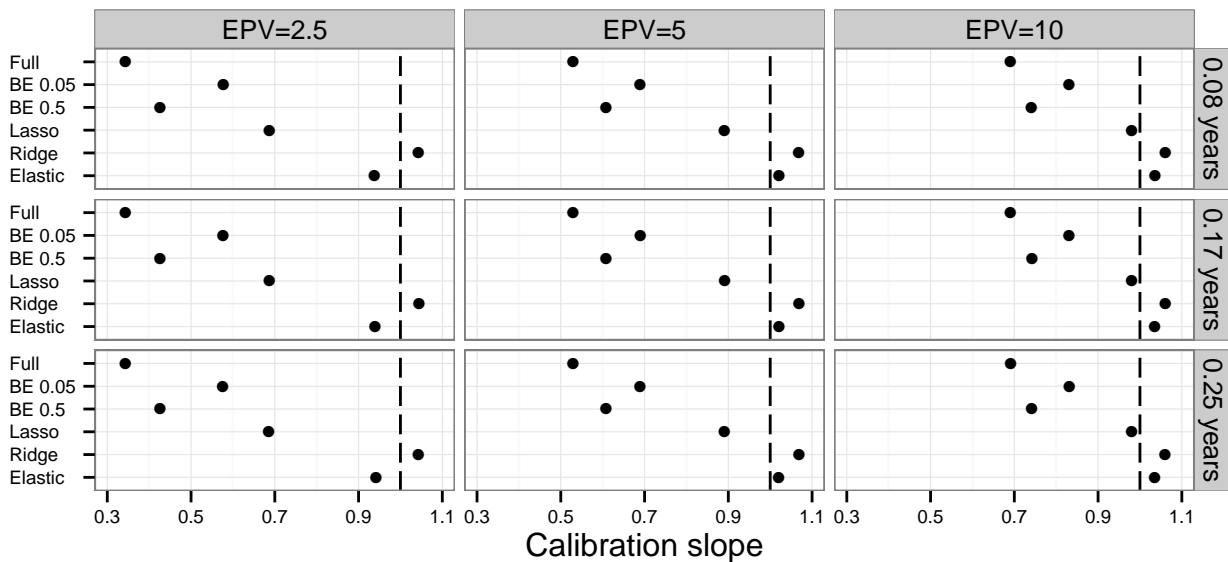**B**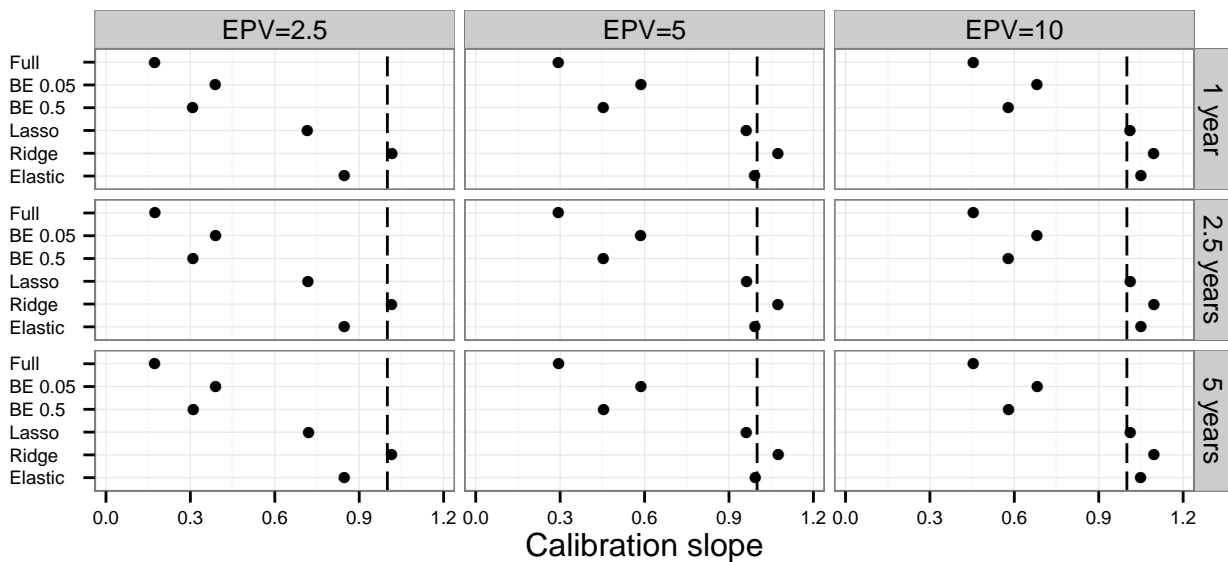

Supplement: Supplementary Figure S2 — Median calibration slope across simulations using different models Median calibration slopes of simulated datasets were calculated using different models in scenario 1 (A) and scenario 2 (B), respectively. Dashed line depicts ideal calibration slope of 1. The models examined include full model, BE with significance levels α = 0.05 and α = 0.5 (BE 0.05 and BE 0.5), ridge, lasso, and elastic net. BE, backward elimination; EPV, events per variable. [file mmc2.pdf]

**A**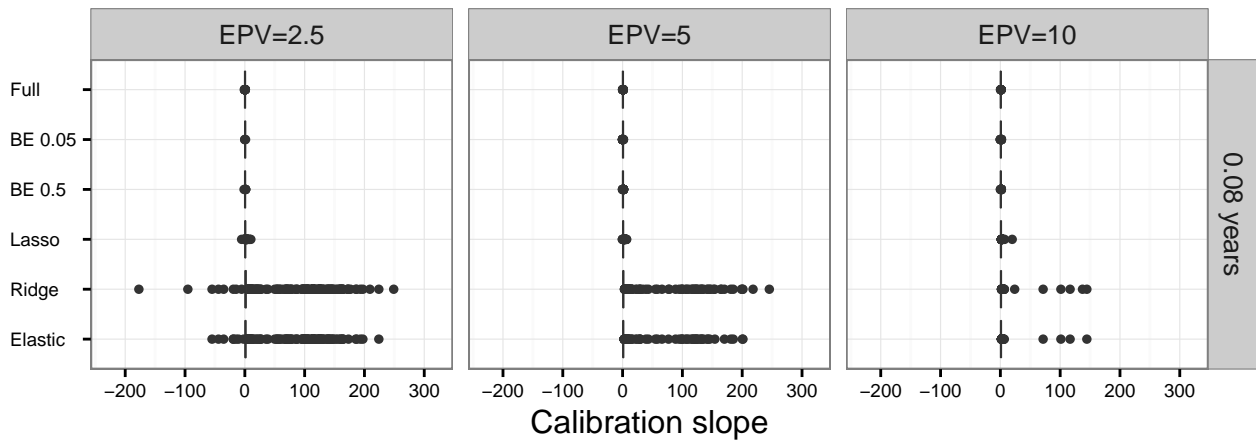**B**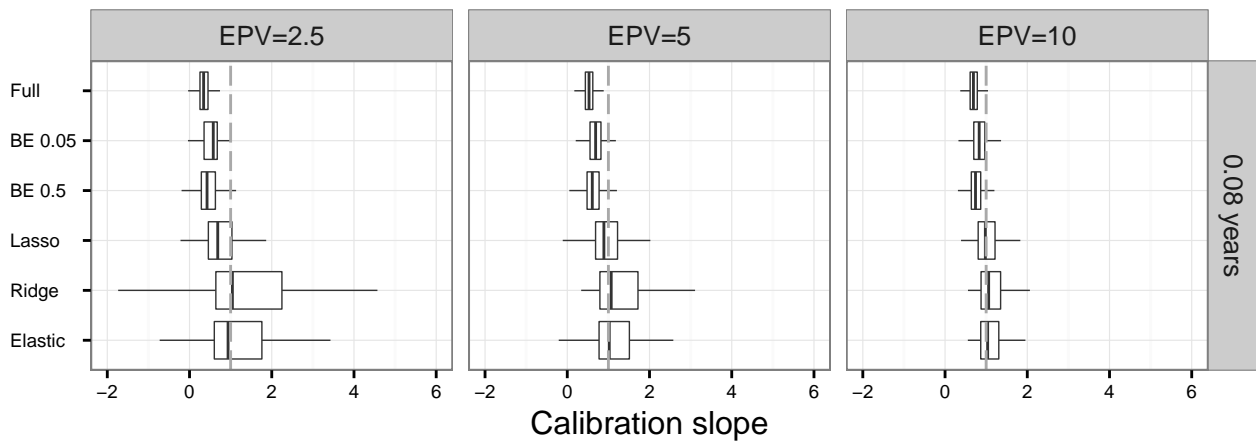

Supplement: Supplementary Figure S3 — Calibration slope distribution across simulations for scenario 1 using different models (A) Boxplots of calibration slopes. (B) Boxplots of calibration slopes with outliers removed to help visualization of quartiles. Predicted event probabilities were computed at time point 0.08 year. Gray dashed line depicts ideal calibration slope of 1. [file mmc3.pdf]

**A**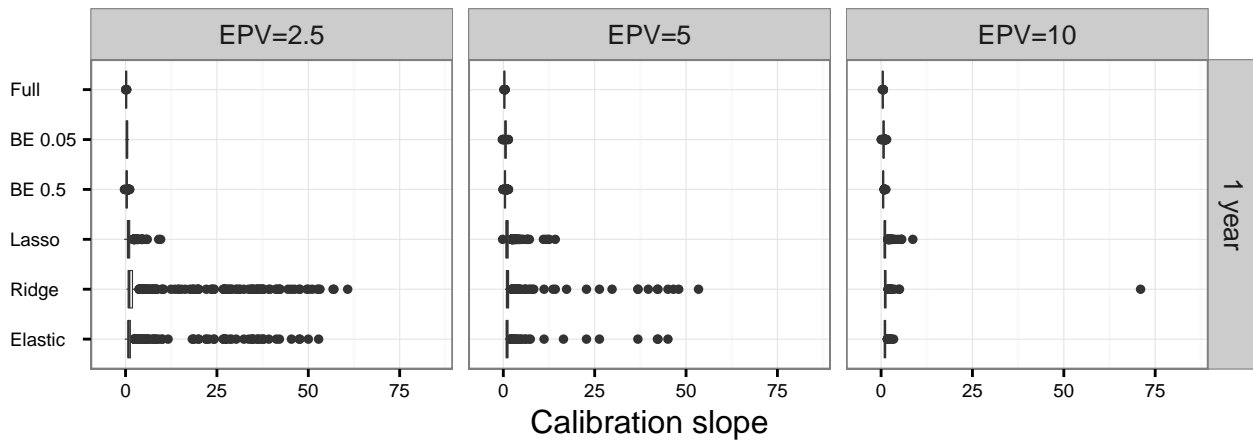**B**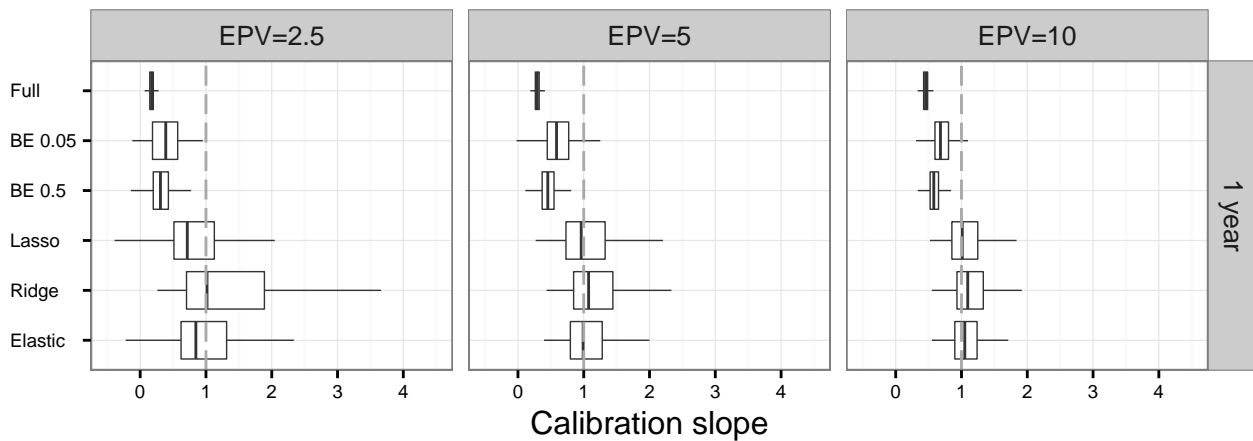

Supplement: Supplementary Figure S4 — Calibration slope distribution across simulations for scenario 2 using different models (A) Boxplots of calibration slopes. (B) Boxplots of calibration slopes with outliers removed to help visualization of quartiles. Predicted event probabilities were computed at time point 1 year. Gray dashed line depicts ideal calibration slope of 1. [file mmc4.pdf]

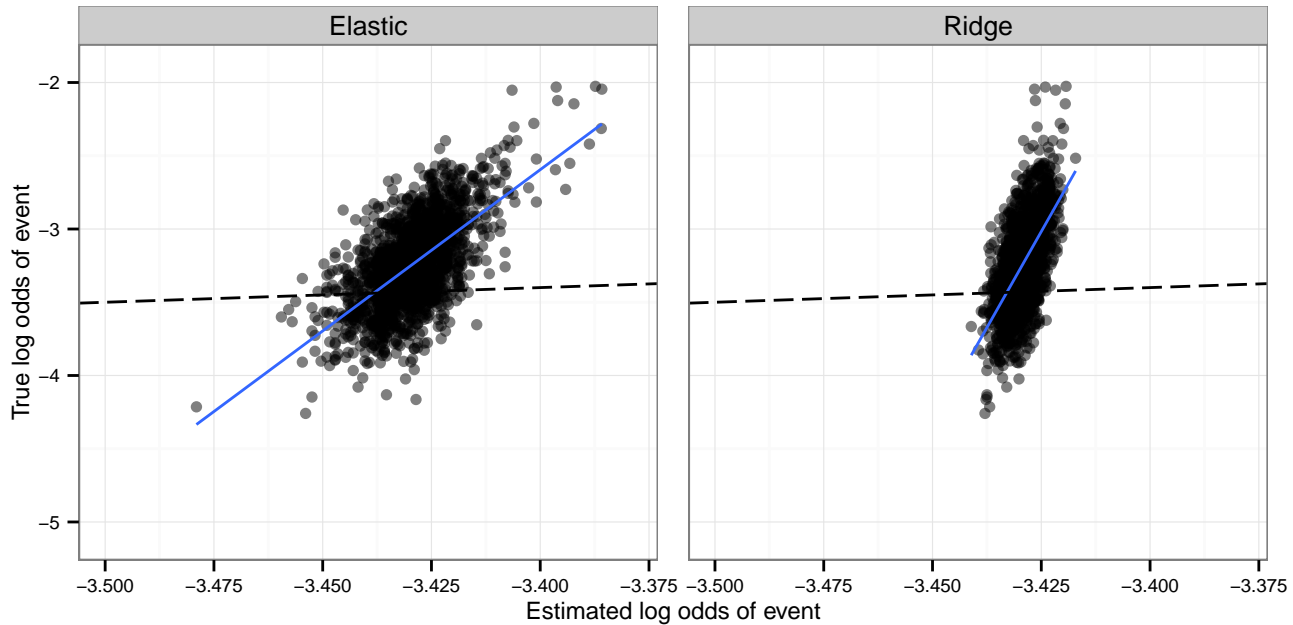

Supplement: Supplementary Figure S5 — Scatter plot of log odds of event for exemplary simulation with extreme calibration slope The true log odds of event was plotted against the estimated log odds of event. Results shown here are for exemplary simulation in scenario 2 with elastic net (left panel) and ridge regression (right panel). One year predicted event probabilities were computed at time point 1 year. Blue line depicts least squares regression line (y = 72.2 + 22 x and y = 176.7 + 52.5 x for elastic net and ridge, respectively). Dashed line depicts the y = x line, which corresponds to the ideal case in which the estimated log odds equals to the true log odds. [file mmc5.pdf]

# A

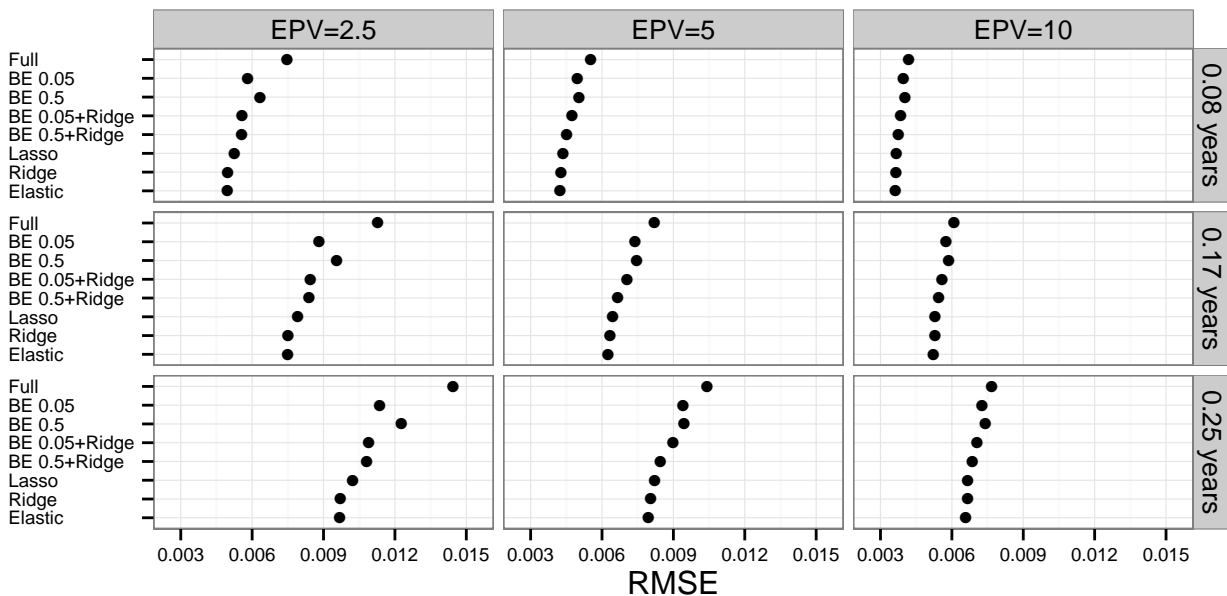

# B

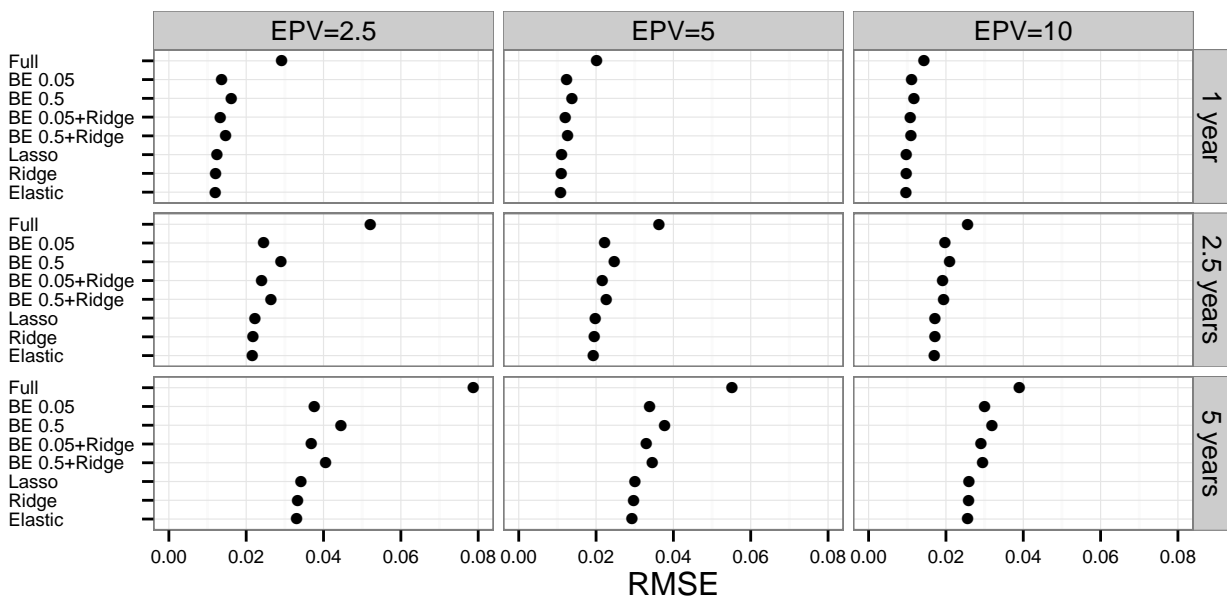

Supplement: Supplementary Figure S6 — Average RMSEs across simulations including application of ridge regression to results of BE Average RMSEs of simulated datasets were calculated using different models in scenario 1 (A) and scenario 2 (B), respectively. Other than full model, BE with significance levels α = 0.05 and α = 0.5 (BE 0.05 and BE 0.5), ridge, lasso, and elastic net, models examined also include application of ridge regression for BE 0.5 (BE 0.5 + Ridge) and BE 0.05 (BE 0.05 + Ridge). Predicted event probabilities were computed at time points 1, 2.5, and 5 years. BE, backward elimination; RMSE, root mean square error; EPV, events per variable. [file mmc6.pdf]

**A**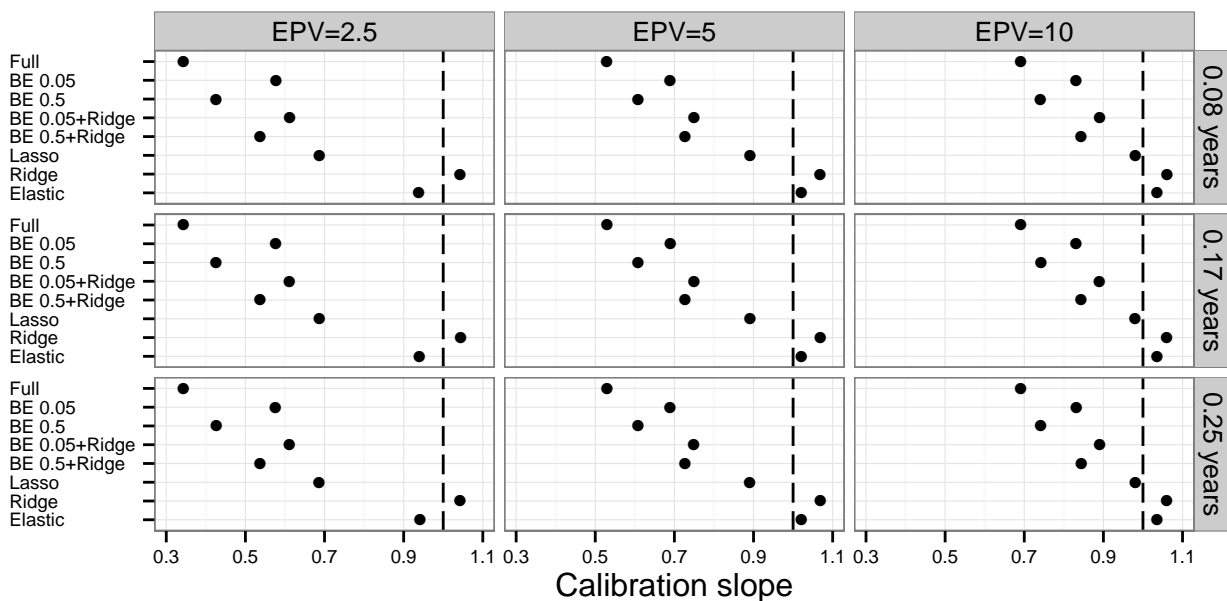**B**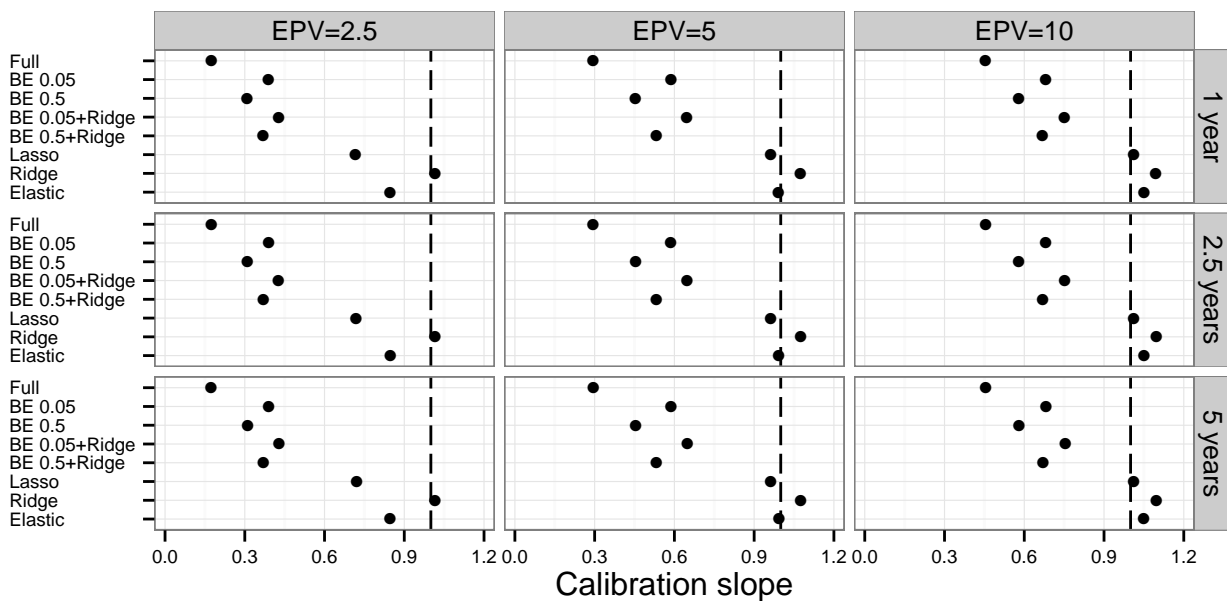

Supplement: Supplementary Figure S8 — Median calibration slopes across simulations including application of ridge regression to results of BE Median calibration slopes of simulated datasets were calculated using different models in scenario 1 (A) and scenario 2 (B), respectively. Dashed line depicts ideal calibration slope of 1. See legend of Figure S6 for more details of the models used and the scenarios examined. [file mmc8.pdf]

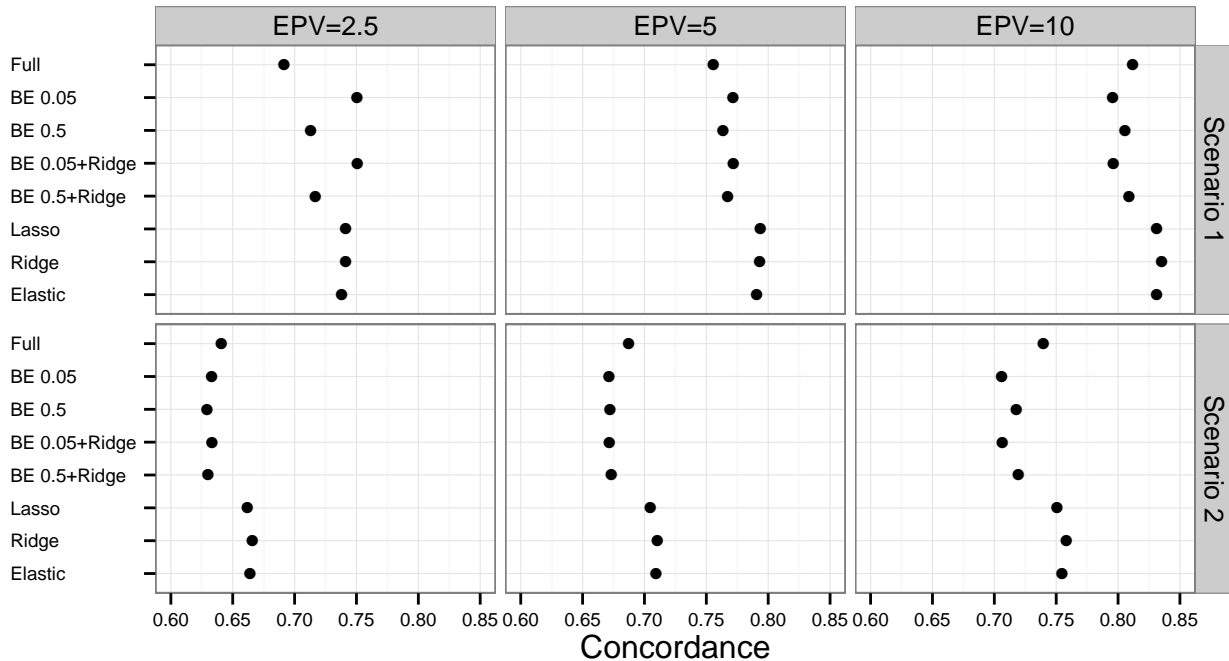

Supplement: Supplementary Figure S9 — Average concordance across simulations including application of ridge regression to results of BE Average concordance of simulated datasets was calculated using different models in scenario 1 (A) and scenario 2 (B), respectively. See legend of Figure S6 for more details of the models used and the scenarios examined. [file mmc9.pdf]
