# Supplementary material for: Comparison of Cox Model Methods in A Low-dimensional Setting with Few Events
Source: Genomics Proteomics Bioinformatics. 2016 May 17;14(4):235–43. doi: 10.1016/j.gpb.2016.03.006 (PMC4996851; doi:10.1016/j.gpb.2016.03.006)
Supplement: Supplementary Table S1 — Baseline characteristics of patients in the AtheroGene study used in our simulations. [file mmc10.docx]

**Table S1 Baseline characteristics of patients of the Athero*Gene* study used in our simulations**

| **Patient characteristics** | **Value** |
| --- | --- |
| Age (years) | 63.0 (55.0, 69.0) |
| Male (%) | 1344 (77.6) |
| Body mass index (kg/m²) | 27.1 (24.9, 29.4) |
| Current smoker (%) | 532 (30.7) |
| Diabetes (%) | 370 (21.4) |
| LDL/HDL cholesterol ratio | 2.9 (2.2, 3.6) |
| Hypertension (%) | 1248 (72.1) |
| Creatinine (mg/dl) | 1.0 (0.9, 1.1) |
| C-reactive protein (mg/l) | 3.9 (1.8, 11.0) |

*Note*: Data were collected from 1731 patients. Continuous variables are given as median (25^th^ percentile, 75^th^ percentile), and categorical ones are presented as number (percentage). HDL, high density lipoprotein; LDL, low density lipoprotein.
