# Supplementary material for: Comparison of Cox Model Methods in A Low-dimensional Setting with Few Events
Source: Genomics Proteomics Bioinformatics. 2016 May 17;14(4):235–43. doi: 10.1016/j.gpb.2016.03.006 (PMC4996851; doi:10.1016/j.gpb.2016.03.006)
Supplement: Supplementary Table S2 — Information on genetic variants considered. [file mmc11.docx]

**Table S2 Information on genetic variants considered**

| **Chromosome** | **SNP/Indel** | **Allele 1** | **Allele 2** | **Frequency allele 1** | **MAF** | ***R^2^*** |
| --- | --- | --- | --- | --- | --- | --- |
| 1 | rs11206510 | T | C | 0.81 | 0.19 | 1.00 |
| 1 | rs6689306 | G | A | 0.56 | 0.44 | 0.94 |
| 1 | rs67180937 | G | T | 0.76 | 0.24 | 0.99 |
| 1 | rs7528419 | A | G | 0.79 | 0.21 | 0.99 |
| 1 | rs9970807 | C | T | 0.93 | 0.07 | 0.97 |
| 2 | chr2:203828796:I | R | I | 0.86 | 0.14 | 0.88 |
| 2 | chr2:21378433:D | D | R | 0.77 | 0.23 | 0.82 |
| 2 | chr2:44074126:D | R | D | 0.74 | 0.26 | 0.85 |
| 2 | rs16986953 | G | A | 0.92 | 0.08 | 0.94 |
| 2 | rs17678683 | T | G | 0.90 | 0.10 | 1.00 |
| 2 | rs7568458 | T | A | 0.55 | 0.45 | 0.98 |
| 3 | chr3:138099161:I | R | I | 0.82 | 0.18 | 0.99 |
| 4 | rs17087335 | G | T | 0.79 | 0.21 | 0.99 |
| 4 | rs4593108 | C | G | 0.86 | 0.14 | 0.98 |
| 4 | rs72689147 | G | T | 0.81 | 0.19 | 1.00 |
| 5 | rs273909 | A | G | 0.89 | 0.11 | 0.85 |
| 6 | rs12202017 | A | G | 0.73 | 0.27 | 0.98 |
| 6 | rs17609940 | G | C | 0.82 | 0.18 | 0.99 |
| 6 | rs4252185 | T | C | 0.93 | 0.07 | 0.38 |
| 6 | rs55730499 | C | T | 0.93 | 0.07 | 0.44 |
| 6 | rs56336142 | T | C | 0.80 | 0.20 | 0.96 |
| 6 | rs9349379 | A | G | 0.58 | 0.42 | 0.82 |
| 7 | rs10953541 | C | T | 0.76 | 0.24 | 1.00 |
| 7 | rs11556924 | C | T | 0.63 | 0.37 | 1.00 |
| 7 | rs2107595 | G | A | 0.82 | 0.18 | 0.92 |
| 7 | rs3918226 | C | T | 0.92 | 0.08 | 0.38 |
| 8 | rs264 | G | A | 0.87 | 0.13 | 0.99 |
| 8 | rs2954029 | A | T | 0.54 | 0.46 | 0.99 |
| 9 | rs2519093 | C | T | 0.77 | 0.23 | 0.96 |
| 9 | rs2891168 | A | G | 0.48 | 0.48 | 1.00 |
| 10 | rs11191416 | T | G | 0.89 | 0.11 | 1.00 |
| 10 | rs1412444 | C | T | 0.66 | 0.34 | 1.00 |
| 10 | rs1870634 | G | T | 0.67 | 0.33 | 0.95 |
| 10 | rs2487928 | G | A | 0.53 | 0.47 | 1.00 |
| 11 | rs10840293 | A | G | 0.59 | 0.41 | 0.92 |
| 11 | rs2128739 | C | A | 0.70 | 0.30 | 1.00 |
| 11 | rs964184 | C | G | 0.84 | 0.16 | 0.99 |
| 12 | rs2681472 | A | G | 0.83 | 0.17 | 0.95 |
| 12 | rs3184504 | C | T | 0.49 | 0.49 | 0.56 |
| 13 | rs11838776 | G | A | 0.70 | 0.30 | 0.99 |
| 13 | rs9319428 | G | A | 0.67 | 0.33 | 1.00 |
| 14 | rs10139550 | C | G | 0.55 | 0.45 | 0.76 |
| 15 | rs17514846 | C | A | 0.50 | 0.50 | 0.91 |
| 15 | rs4468572 | C | T | 0.58 | 0.42 | 1.00 |
| 15 | rs56062135 | C | T | 0.78 | 0.22 | 0.97 |
| 15 | rs8042271 | G | A | 0.96 | 0.04 | 0.90 |
| 17 | rs12936587 | G | A | 0.57 | 0.43 | 0.98 |
| 17 | rs216172 | G | C | 0.64 | 0.36 | 0.98 |
| 17 | rs46522 | T | C | 0.55 | 0.45 | 0.96 |
| 17 | rs7212798 | T | C | 0.83 | 0.17 | 0.91 |
| 18 | rs663129 | G | A | 0.75 | 0.25 | 0.99 |
| 19 | rs4420638 | A | G | 0.81 | 0.19 | 1.00 |
| 19 | rs56289821 | G | A | 0.90 | 0.10 | 0.27 |
| 21 | rs28451064 | G | A | 0.86 | 0.14 | 0.94 |
| 22 | rs180803 | G | T | 0.99 | 0.01 | 0.88 |

*Note: R^2^* (R squared) is an imputation quality measure that estimates the squared correlation between the allele counts of the true and imputed genotypes. Insertions and deletions are indicated with “I” and “D”, respectively. MAF, minor allele frequency.
