# Supplementary material for: Comparison of Cox Model Methods in A Low-dimensional Setting with Few Events
Source: Genomics Proteomics Bioinformatics. 2016 May 17;14(4):235–43. doi: 10.1016/j.gpb.2016.03.006 (PMC4996851; doi:10.1016/j.gpb.2016.03.006)
Supplement: Supplementary Table S3 — Regression coefficients for scenario 1 (EPV = 23.22) using different models. [file mmc12.docx]

**Table S3 Regression coefficients for scenario 1 (EPV = 23.22) using different models**

|  | **Full** | **BE 0.05** | **BE 0.5** | **Lasso** | **Ridge** | **Elastic** |
| --- | --- | --- | --- | --- | --- | --- |
| Age | 0.0167 |  | 0.0194 | 0.0142 | 0.0149 | 0.0141 |
| Male | −0.2325 |  |  | −0.1553 | −0.1600 | −0.1443 |
| Body mass index | −0.0016 |  |  |  | −0.0009 |  |
| Current smoker | 0.1175 |  |  | 0.0487 | 0.0939 | 0.0526 |
| Diabetes | 0.2219 |  |  | 0.1849 | 0.2122 | 0.1883 |
| LDL/HDL cholesterol ratio | 0.0134 |  |  |  | 0.0153 |  |
| Hypertension | 0.0078 |  |  |  | 0.0264 |  |
| Log(creatinine, mg/dl) | 1.8373 | 2.0446 | 1.8200 | 1.7506 | 1.4961 | 1.6655 |
| Log(C-reactive protein, mg/l) | 0.0860 |  | 0.0959 | 0.0727 | 0.0755 | 0.0717 |

*Note*: Scenario 1 candidate predictors include clinical variables and biomarkers. The coefficients represent the weights given to each predictor variable by the regression model. BE, backward elimination; EPV, events per variable; HDL, high density lipoprotein; LDL, low density lipoprotein.
