# Supplementary material for: Comparison of Cox Model Methods in A Low-dimensional Setting with Few Events
Source: Genomics Proteomics Bioinformatics. 2016 May 17;14(4):235–43. doi: 10.1016/j.gpb.2016.03.006 (PMC4996851; doi:10.1016/j.gpb.2016.03.006)
Supplement: Supplementary Table S4 — Regression coefficients for scenario 2 (EPV = 3.27) using different models. [file mmc13.docx]

**Table S4 Regression coefficients for scenario 2 (EPV = 3.27) using different models**

| **Variable** | **Full** | **BE 0.05** | **BE 0.5** | **Lasso** | **Ridge** | **Elastic** |
| --- | --- | --- | --- | --- | --- | --- |
| Age | 0.0184 |  | 0.0207 | 0.0130 | 0.0088 | 0.0122 |
| Male | −0.2217 |  |  | −0.0677 | −0.0516 | −0.0438 |
| Body mass index | –0.0046 |  |  |  | –0.0002 |  |
| Current smoker | 0.0884 |  |  |  | 0.0403 |  |
| Diabetes | 0.1816 |  |  | 0.1257 | 0.1320 | 0.1296 |
| LDL/HDL cholesterol ratio | –0.0152 |  |  |  | 0.0079 |  |
| Hypertension | –0.0617 |  |  |  | 0.0275 |  |
| Log(creatinine, mg/dl) | 1.9051 | 2.0446 | 1.8616 | 1.6311 | 0.7334 | 1.4352 |
| Log(C-reactive protein, mg/l) | 0.1011 |  |  | 0.0575 | 0.0432 | 0.0535 |
| rs11206510 | 0.2264 |  |  | 0.0529 | 0.0511 | 0.0387 |
| rs9970807 | –0.3460 |  |  | –0.2038 | –0.1403 | –0.1799 |
| rs7528419 | 0.0986 |  |  |  | 0.0317 |  |
| rs6689306 | –0.0538 |  |  |  | –0.0233 |  |
| rs67180937 | 0.1105 |  |  |  | 0.0288 |  |
| rs16986953 | –0.0084 |  |  |  | –0.0188 |  |
| chr2:21378433:D | 0.1868 |  |  | 0.0245 | 0.0508 | 0.0192 |
| chr2:44074126:D | –0.1058 |  |  | –0.0097 | –0.0482 | –0.0116 |
| rs7568458 | –0.1486 |  |  | –0.0580 | –0.0525 | –0.0496 |
| rs17678683 | 0.0128 |  |  |  | 0.0028 |  |
| chr2:203828796:I | 0.2026 |  |  | 0.0479 | 0.0772 | 0.0455 |
| chr3:138099161:I | –0.0159 |  |  |  | –0.0071 |  |
| rs4593108 | –0.0225 |  |  |  | –0.0199 |  |
| rs72689147 | 0.2467 |  |  | 0.0769 | 0.0727 | 0.0658 |
| rs273909 | 0.3064 |  |  | 0.1233 | 0.1087 | 0.1101 |
| rs9349379 | –0.0417 |  |  |  | –0.0097 |  |
| rs17609940 | –0.0972 |  |  |  | –0.0253 |  |
| rs56336142 | 0.0250 |  |  |  | 0.0049 |  |
| rs12202017 | 0.1547 |  |  | 0.0495 | 0.0547 | 0.0427 |
| rs55730499 | 0.1417 |  |  |  | 0.1329 | 0.0375 |
| rs4252185 | 0.5089 |  |  | 0.2651 | 0.1683 | 0.2020 |
| rs2107595 | –0.0348 |  |  |  | –0.0170 |  |
| rs10953541 | 0.0408 |  |  |  | 0.0133 |  |
| rs11556924 | –0.0368 |  |  |  | –0.0112 |  |
| rs264 | 0.0837 |  |  |  | 0.0340 |  |
| rs2954029 | –0.0222 |  |  |  | –0.0201 |  |
| rs2891168 | –0.0108 |  |  |  | 0.0017 |  |
| rs2519093 | –0.2501 |  |  | –0.1153 | –0.0860 | –0.1010 |
| rs2487928 | –0.0395 |  |  |  | –0.0074 |  |
| rs1870634 | 0.0448 |  |  |  | 0.0072 |  |
| rs1412444 | –0.0280 |  |  |  | –0.0128 |  |
| rs11191416 | 0.2012 |  |  | 0.0010 | 0.0564 |  |
| rs2128739 | 0.0017 |  |  |  | 0.0177 |  |
| rs964184 | –0.0865 |  |  |  | –0.0051 |  |
| rs2681472 | 0.0578 |  |  |  | 0.0285 |  |
| rs3184504 | –0.0710 |  |  |  | –0.0208 |  |
| rs9319428 | –0.0265 |  |  |  | 0.0106 |  |
| rs11838776 | –0.0374 |  |  |  | –0.0122 |  |
| rs10139550 | –0.2105 |  |  | –0.1098 | –0.0795 | –0.0959 |
| rs4468572 | 0.2565 |  |  | 0.1357 | 0.0812 | 0.1157 |
| rs17514846 | –0.2018 |  |  | –0.0721 | –0.0570 | –0.0592 |
| rs216172 | –0.0973 |  |  |  | –0.0251 |  |
| rs12936587 | 0.1282 |  |  | 0.0342 | 0.0457 | 0.0291 |
| rs46522 | –0.0240 |  |  |  | –0.0082 |  |
| rs56289821 | –0.0784 |  |  |  | –0.0554 |  |
| rs4420638 | –0.2077 |  |  | –0.0866 | –0.0726 | –0.0757 |
| rs28451064 | 0.0895 |  |  |  | 0.0304 |  |
| rs17087335 | 0.0275 |  |  |  | –0.0031 |  |
| rs3918226 | 0.3637 |  |  | 0.0809 | 0.1279 | 0.0679 |
| rs10840293 | 0.1413 |  |  | 0.0119 | 0.0417 | 0.0106 |
| rs56062135 | 0.0402 |  |  |  | 0.0007 |  |
| rs8042271 | –0.1445 |  |  |  | –0.0807 |  |
| rs7212798 | 0.0363 |  |  |  | 0.0060 |  |
| rs663129 | –0.0411 |  |  |  | –0.0122 |  |
| rs180803 | 0.2235 |  |  |  | 0.0945 |  |

*Note*: Scenario 2 candidate predictors include clinical variables and biomarkers, as well as genetic variants. The coefficients represent the weights given to each predictor variable by the regression model. BE, backward elimination; EPV, events per variable; HDL, high density lipoprotein; LDL, low density lipoprotein.
