# Supplementary material for: Comparison of Cox Model Methods in A Low-dimensional Setting with Few Events
Source: Genomics Proteomics Bioinformatics. 2016 May 17;14(4):235–43. doi: 10.1016/j.gpb.2016.03.006 (PMC4996851; doi:10.1016/j.gpb.2016.03.006)
Supplement: Supplementary Figure S7 — Average calibration slopes across simulations including application of ridge regression to results of BE Average calibration slopes of simulated datasets were calculated using different models in scenario 1 (A) and scenario 2 (B), respectively. Dashed line depicts ideal calibration slope of 1. See legend of Figure S6 for more details of the models used and the scenarios examined. [file mmc7.pdf]

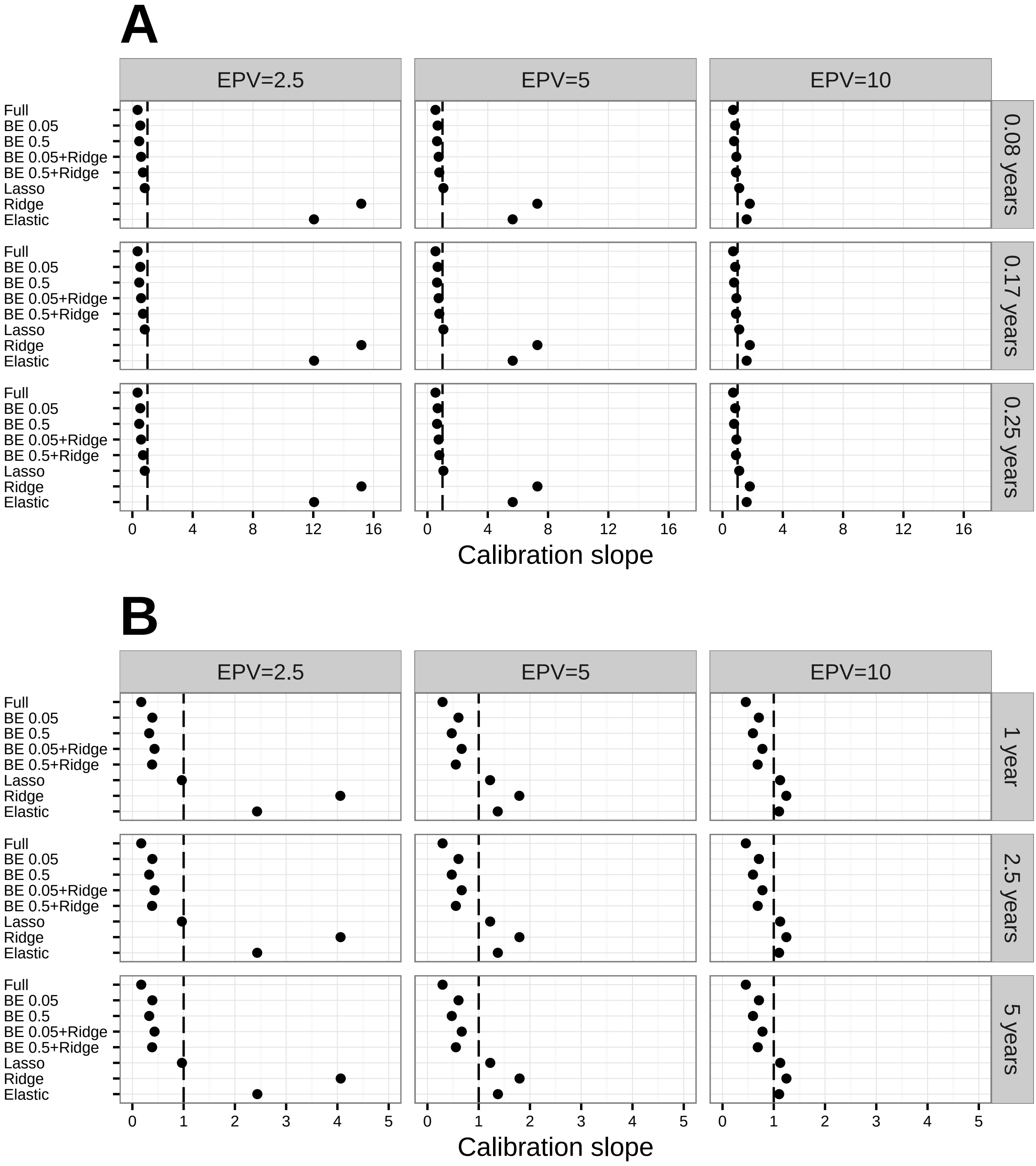
A
EPV=2.5
EPV=5
EPV=10
Full
BE 0.05
BE 0.5
BE 0.05+Ridge
BE 0.5+Ridge
Lasso
Ridge
Elastic
0.08 years
0.17 years
0.25 years
0
4
8
12
16
Calibration slope
B
EPV=2.5
EPV=5
EPV=10
1 year
2.5 years
5 years
0
1
2
3
4
5
Calibration slope
